# The Use and Abuse of the Stomach Tube*Read before the Brazos Valley Medical Society, May Meeting, 1906.

**Published:** 1906-07

**Authors:** J. W. Torbett

**Affiliations:** Marlin, Texas


For Texas Medical Journal.

### The Use and Abuse of the Stomach Tube.*

BY J. W. TORBETT, M. D., MARLIN, TEXAS.

The stomach tube is a means of doing much good if rightly used, but its promiscuous use in all classes of stomach troubles without a definite knowledge of the indications calling for its proper and beneficial use prompts me to write this article. Some physicians

*Read before the Brazos Valley Medical Society, May Meeting, 1906.

use the tube too much, while others never use it all; so, in this article I shall endeavor to point out and classify the indications for its use, which a rather large and varied study and experience of eight years in treating stomach troubles have taught me. I recognize the fact that eminent authorities differ quite frequently in the treatment of the same diseases. I recognize, also, with equal force that the patient's temperament and the physician's personality and judgment in applying the treatment are great factors that figure in all results obtained. Of course, it is best that I state in this article that I give the results of my experience in the application of certain lines of treatment, for which I claim no credit, however, as originator, only a few modifications in technique, which will be explained and illustrated hereafter.

### ORIGIN OF TUBE.

The credit is due Dr. Philip S. Physick, of the University of Pennsylvania, for the invention and practical use of the stomach tube in about 1800. It was first used for removing poisons from the stomach. In 1867 it was first used with success by Kussmaul for therapeutic purposes, especially in gastric dilatation; and in 1871 was recommended by Leube for diagnostic purposes, thereby making possible since 1880 the correct diagnosis and treatment of gastro-intestinal diseases.

In selecting a tube it is best to choose one with a bulb, medium size, moderately stiff, with no sharp edges or cracks near the end to injure the mucous membrane. The tube should be thoroughly washed and disinfected after use and the same tube used only on the same patient so long as treatment is needed.

### USES.

One of the most important uses of the tube is the removal of poisons which have been recently taken into the stomach. It can be used at once, before an emetic could have time to act, and the stomach repeatedly washed out until all traces are removed. It is best, however, to give an antidote before the tube is inserted. In one case of iodin poisoning in which I was called, I ordered starch water to be given at once before I arrived with the tube, and when the stomach was washed out the poison was entirely neutralized. In the same way I have ordered whisky or alcohol to be administered in a case where carbolic acid was taken by mistake.

Another important use of the tube is for diagnostic purposes.

All cases of stomach trouble that have continued for a few weeks and that do not get better by ordinary diet or treatment, or that steadily grow worse, should be examined by means of the stomach tube.

The tube is introduced best by having the patient slightly elevate his chin, open his mouth, the end of the tube thoroughly wet in warm water, is passed back to the pharynx, the patient told to close his lips, breathe deeply through his nose and swallow, the tube is gently pushed into the stomach. A slight resistance is usually met at the cardiac entrance. The introduction of the tube shows: (1) The condition of the œsophagus, whether strictured or not; (2) by forcing air into the stomach through the bifurcated tube the percussion method will usually determine with readiness the size of the stomach, and the escape of the air through the whistle on the bifurcation not connected with the compressed air apparatus will show the contractility and motor power of the stomach much better, I think, than the use of iodid of potash or iodipin, as frequently recommended; (3) it serves to remove the test meal for chemical examination, and thereby makes a diagnosis possible which could not otherwise be made. In removing the test meal it is frequently much more easily done by having the patient recline on the left side with head some lower than the body, after the tube is in position, thereby securing the aid of gravity, as well as the suction of the bulb. This question can not be dealt with here, but will say in passing, that the test for HCl acidity, butyric, and lactic acids and the microscopical examinations can be made quickly by most anyone and will be frequently all the chemical examination needed.

Not more than a pint of warm water should be introduced into the stomach and withdrawn by siphon and the quantity measured. If this be done before breakfast, as it should, and the amount be more than that introduced, it shows retention or hypersecretion, with or without stagnation, which calls for the continued use of the tube as a *therapeutic agent.*

There can be no doubt about the beneficial use of the tube in washing out those cases in which there is retention and fermentation found before breakfast in the morning. A stomach which never empties itself never rests. It is frequently best in such cases to wash it out about four hours after a light supper and let it rest during the night. The retention may be due to pyloric obstruction, which, if organic, can not be relieved by lavage, of course, but the fermentation may be relieved and the patient's health benefited. If, however, the retention be due to myasthenia, as is usually the case with or without dilatation, the washings and the medicated air after the method of Turck should be used, the frequency to be determined by the effects, some few cases requiring washing night and morning, some not oftener than every second day. In those cases of a nervous hypersensitive mucous membrane, causing nausea easily, every second day is usually best. This method is not generally used, but in that class of cases the results are simply marvelous. Those cases of myasthenia with dilatation or motor insufficiency are very frequently diagnosed as liver trouble or malaria, and go the rounds with all sorts of treatment and no benefit. They frequently do not even suspect that stomach trouble is the cause, but rather think it is the result of their disease. I have had several such cases so emaciated they could scarcely walk alone; after three to six weeks' treatment regain their former health and vigor. The contractility of the stomach in those cases was so small that it would not force the air out of the stomach sufficiently strong to sound the small whistle connected in the tube. Turck's gyromele is a very good method of determining the size and shape of the stomach, but as I have been able to determine that by means of air and percussion, and as I lost the sponge in a patient's stomach when I first began its use, it has not been a favorable method of mine.

The water used should be at the temperature of 120° F., and for cleansing purposes should have bicarbonate of soda and salt; for allaying secretion and irritability a solution of 1/2000 to 1/5000 nitrate of silver should be used; and as a disinfectant 1/5000 permanganate of potash or a solution of resorcin.

So the tube should be used in cases: (1) With retention or stagnation of food and the digestive products in stomach or œsophagus; (2) retention of excessive secretions, as in gastrosuccorrhea or Reichman's disease; (3) for the severe chronic cases of gastritis with excessive mucous secretion; (4) in gastric neurosis for psychic effect; (5) in all cases where there is excessive and active germ growth, as indicated by fermentation and the microscope; (6) in diseases of the œsophagus where there is stricture, as food can not otherwise reach the stomach; (7) in the removal of poisons ingested; (8) the pneumatic gymnastic should be used in connection with these cases of dilatation and myasthenia.

ABUSE.

The tube should not be used unless there are some indications mentioned above calling for its use. Neither should it be used in: (1) Severe heart diseases in which the patient is very liable to faint; (2) aneurism of the large arteries and in all cases of recent hemorrhage from whatever cause; (3) severe ulcers of the stomach; (4) tendency to apoplexy if nausea is easily excited, as that would raise arterial tension and might induce hemorrhage in the brain. I have seen some cases of extreme weakness and dizziness, however, caused by auto-intoxication in which the tube did great good. Such cases had at first been diagnosed as threatened apoplexy.

